# Substandard urological care of elderly patients with spinal cord injury: an unrecognized epidemic?

**DOI:** 10.1186/1754-9493-8-4

**Published:** 2014-01-21

**Authors:** Subramanian Vaidyanathan, Gurpreet Singh, Bakul Soni, Peter Hughes, Kamesh Pulya

**Affiliations:** 1 Regional Spinal Injuries Centre, Southport and Formby District General Hospital, Town Lane, Southport, Merseyside PR8 6PN, UK; 2 Department of Urology, Southport and Formby District General Hospital, Town Lane, Southport, Merseyside PR8 6PN, UK; 3 Department of Radiology, Southport and Formby District General Hospital, Town Lane, Southport, Merseyside PR8 6PN, UK; 4 Department of Cardiology, Southport and Formby District General Hospital, Town Lane, Southport, Merseyside PR8 6PN, UK

**Keywords:** Spinal cord injury, Elderly patients, Substandard care

## Abstract

**Background:**

We report the anecdotal observation of substandard urological care of elderly paraplegic patients in the community suffering from long-term sequelae of spinal cord injuries. This article is designed to increase awareness of a problem that is likely underreported and may represent the ‘tip of the iceberg’ related to substandard care provided to the vulnerable population of elderly patients with chronic neurological impairment.

**Findings:**

A registered Nurse changed the urethral catheter of an 80-year-old-male with paraplegia; patient developed profuse urethral bleeding and septicaemia. Ultrasound revealed balloon of Foley catheter located in membranous urethra. Flexible cystoscopy was performed and a catheter was inserted over a guide wire. Urethral bleeding recurred 12 days later. This patient was discharged after protracted stay in spinal unit. A nurse changed urethral catheter in an 82-year-old male with paraplegia. The catheter did not drain urine; patient developed pain in lower abdomen. The balloon of Foley catheter was visible behind the urethral meatus, which indicated that the balloon had been inflated in penile urethra. The catheter was removed and a 16 French Foley catheter was inserted per urethra. About 1300 ml of urine was drained. A 91-year-old lady with paraplegia underwent routine ultrasound examination of urinary tract by a Consultant Radiologist, who reported a 4 cm × 3 cm soft tissue mass in the urinary bladder. Cystoscopy was performed without anaesthesia in lithotomy position. Cystoscopy revealed normal bladder mucosa; no stones; no tumour. Following cystoscopy, the right knee became swollen and there was deformity of lower third of right thigh. X-ray revealed fracture of lower third of right femur. Femoral fracture was treated by immobilisation in full plaster cast. Follow-up ultrasound examination of urinary tract, performed by a senior Radiologist, revealed normal outline of urinary bladder with no tumour or calculus.

**Conclusion:**

The adverse outcomes can be averted if elderly spinal cord injury patients are treated by senior, experienced health professionals, who are familiar with changes in body systems due to old age, compounded further by spinal cord injury.

## Background

### Do elderly patients get substandard health care?

Bergman and associates assessed quality of surgical care delivered to 143 consecutive patients 65 years or older, undergoing elective major abdominal surgery at a single university-affiliated hospital in Canada [1]. Adherence to 15 process-based quality indicators was measured, and a pass rate was calculated for each individual quality indicators. Quality of care delivered to elderly patients undergoing major surgery was generally poor and independent of patient characteristics. Quality indicators with the lowest pass rates included postoperative delirium screening (0%), level of care documentation (0.7%), cognition and functional assessment at discharge (4.9%), oral intake documentation (12.6%), and pressure ulcer risk assessment (35.0%).

### Elderly patients need to be treated by senior, experienced doctors

We wish to highlight the importance of clinical experience of doctors and nurses in providing optimum medical treatment especially to elderly spinal cord injury patients. Significant decreases in operative time, length of intubation, and hospital stay were seen as experience of the surgeon in transoral robotic surgery for head and neck tumors increased. Over a four-year period, the mean operative time decreased by 47%; total mean intubation time decreased by 87% and mean hospital stay decreased from 3.0 days to 1.4 days [2]. We report three elderly spinal cord injury patients, who received sub-optimal urological care in the community or in a district general hospital, although care was delivered by qualified staff. These cases illustrate the need for elderly spinal cord injury patients to be treated by senior, experienced health professionals, who will be able to recognise the alterations in urinary tract or skeletal system due to chronic spinal cord injury, which are compounded by old age. Unfamiliarity predisposes to complications after routine procedures such as urethral catheterisation or positioning of lower limbs in lithotomy for cystoscopy. Similarly, failure to comprehend the changes in neuropathic bladder can lead to misdiagnosis during routine ultrasound examination, as indeed happened to one of these patients.

### Duties of a doctor when substandard care is delivered

We discuss duties of a doctor following adverse outcomes. In the United Kingdom, the “Good Medical Practice Guidance” publication developed by the General Medical Council outlines the professional requirements of doctors practising in the United Kingdom when adverse outcomes occur [3]. Doctors must be open and honest with patients if things go wrong. If a patient has suffered harm or distress, the doctor should put matters right, if that is possible; offer an apology; explain fully and promptly what has happened and the likely short-term and long-term effects. The National Health Service in England, United Kingdom commits to ensure that when mistakes happen while receiving health care, patients receive an appropriate explanation and apology, delivered with sensitivity and recognition of the trauma the patient has experienced, and know that lessons will be learned to help avoid a similar incident occurring again [4]. Barraclough has specified principles of greater transparency and open disclosure after an adverse outcome. Firstly, when unintended harm occurs, it involves informing patients and carers about what has gone wrong in an empathic way that includes an expression of regret. Secondly, it involves in depth analysis of the problem, including root cause analysis for severe problems. Thirdly, it involves a commitment by individual (doctors) and organisations to fix the problems identified [5].

### Case reports

#### Patient # 1

**Figure 1:**
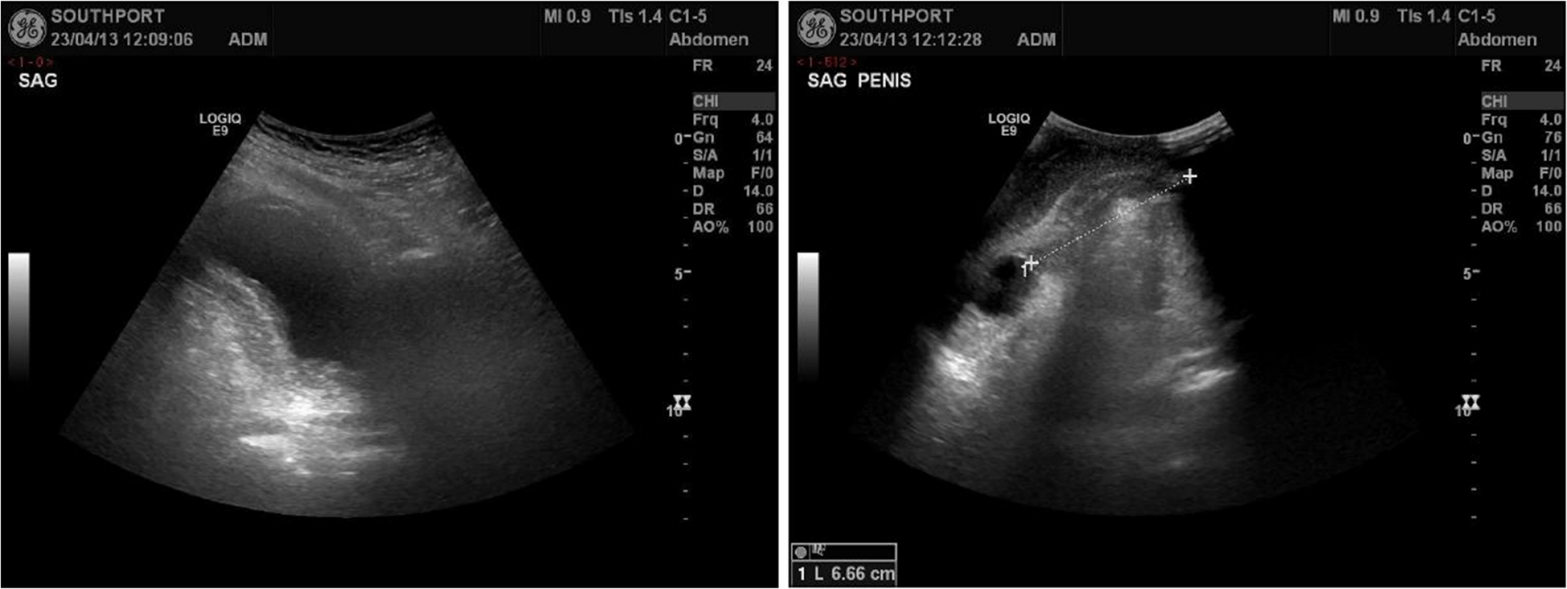
**Top panel: Ultrasound scan of patient # 1 revealed no urinary catheter in the bladder.** Bottom panel: The balloon of Foley catheter was seen in membranous urethra, 7 cm from the tip of penis.

An 80 year-old male, had undergone thoracolumbar decompression for spinal stenosis and developed L-2 complete paraplegia. This patient was transferred to spinal unit for rehabilitation. He had an indwelling urethral catheter. This patient developed temperature and a drop in oxygen saturation. Clinical diagnosis was pneumonia. Chest X-ray revealed hyper inflated chest; lungs were clear with no active lesions; maintained cardiothoracic ratio. Urine culture showed growth of *Pseudomonas aeruginosa* sensitive to Meropenem. This patient was prescribed Meropenem intravenously. Subsequently, this patient developed severe paraphimosis and circumcision was performed. Histology revealed mild non-specific chronic inflammation. There was no evidence of neoplasia. While this patient stayed in a rehabilitation facility, urethral catheter was changed by a Registered Nurse. Following catheterisation, he developed profuse bleeding per urethra and high temperature. Urgent ultrasound examination revealed no urinary catheter in the bladder. (Figure 1) The balloon of Foley catheter was seen in membranous urethra, 7 cm from the tip of penis. (Figure 1) Flexible cystoscopy was performed; bleeding was seen to arise from the site where the balloon of Foley catheter had been inflated. A 16 French Foley catheter was inserted over a 0.032” guide wire. This patient received Meropenem one gram every eight hours intravenously. White cell count was 17.7. Neutrophil: 16.6. Urea: 8.2 mmol/L. Creatinine: 87 umol/L. C-reactive protein: 82.0 mg/L. Random glucose: 15.5 mmol/L. Lactate: 7.6 mmol/L. Urine culture showed coliform species sensitive to amoxicillin and gentamicin. Blood culture yielded *Proteus mirabilis* sensitive to amoxicillin and gentamicin. This patient was prescribed amoxicillin 2 grams every eight hours intravenously. His condition improved. However, twelve days later, this patient again developed profuse bleeding per urethra; bleeding subsided following prolonged local compression over perineum. Subsequently, this patient required exchange of urethral catheter over a 0.032” guide wire. Therefore, this patient needed ambulance to bring him to spinal unit every four weeks for change of urethral catheter.

#### Patient # 2

**Figure 2:**
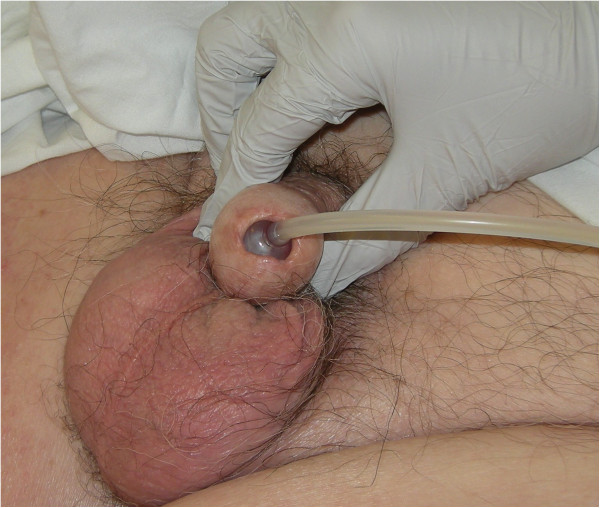
**Clinical photograph of penis of patient # 2: The balloon of Foley catheter is visible through the urethral meatus.** The balloon has been inflated in distal penile urethra.

An 82-year-old male underwent decompression at T-11/12 for spinal stenosis, four years previously in 2008 because of pain and weakness in lower limbs. He was walking with two walking canes before the operation and did not have problem with bladder and bowel control. However, after surgery, this patient could not move or feel his legs at all. Urgent MRI revealed extradural haematoma with compression of the spinal cord at T-11 and T-12 levels. This patient underwent revision of decompression of T-10 to T-12 and evacuation of blood clot. The second operation did not produce recovery of his motor power and sensation in lower limbs. This patient also lost control of his bowels and urinary bladder. This patient was managing his bladder by indwelling urethral catheter; catheter was changed by a District Nurse. In 2011, urethral catheter got blocked and the catheter was changed during night by a Registered Nurse. The catheter did not drain urine. This patient was getting pain in lower abdomen. This patient attended spinal unit in the morning. On clinical examination, an unusually long segment of Foley catheter was lying outside the penis. The balloon of Foley catheter could be palpated in distal penile urethra. On close inspection, the balloon of Foley catheter was just visible behind the urethral meatus. (Figure 2) The balloon was deflated. A 16 French Foley catheter was inserted per urethra. About 1300 ml of urine was drained. This patient developed profuse haematuria, which subsided over the next 48 hours. Subsequently, this patient preferred to come to spinal unit for change of urethral catheter, as he and his wife lost trust in the community health care professionals for changing urethral catheter. This patient required ambulance to bring him to spinal unit every fortnight for change of urethral catheter.

#### Patient # 3

**Figure 3:**
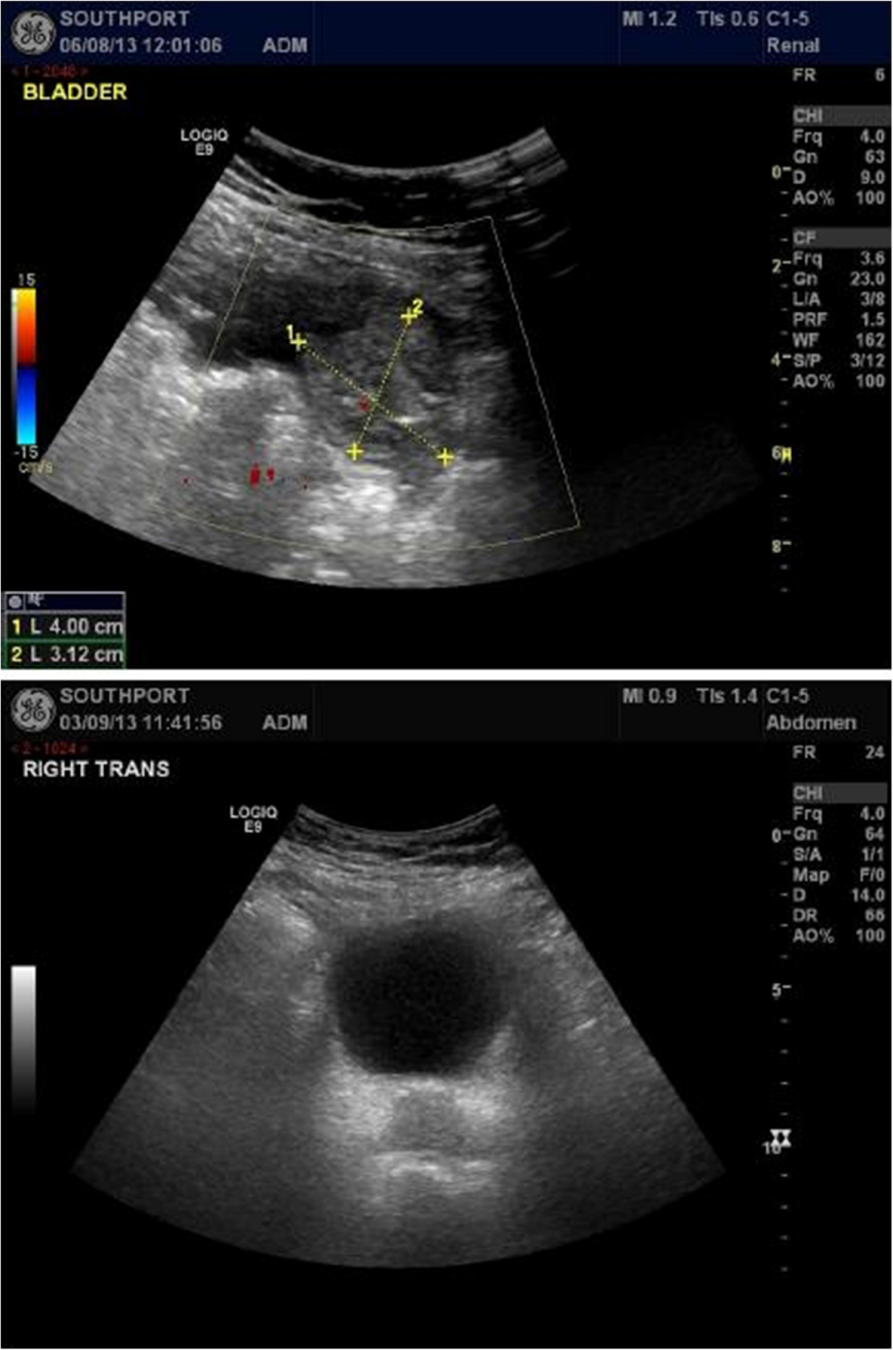
**Top panel: Ultrasound scan of urinary bladder of patient # 3 was reported to show a large 4 cm × 3 cm soft tissue mass in the urinary bladder.** Bottom panel: Follow-up ultrasound examination of urinary bladder performed by a senior Radiologist: Suprapubic catheter was in right position. Outline of urinary bladder was normal; there was no neoplastic filling defect or calculus.

**Figure 4:**
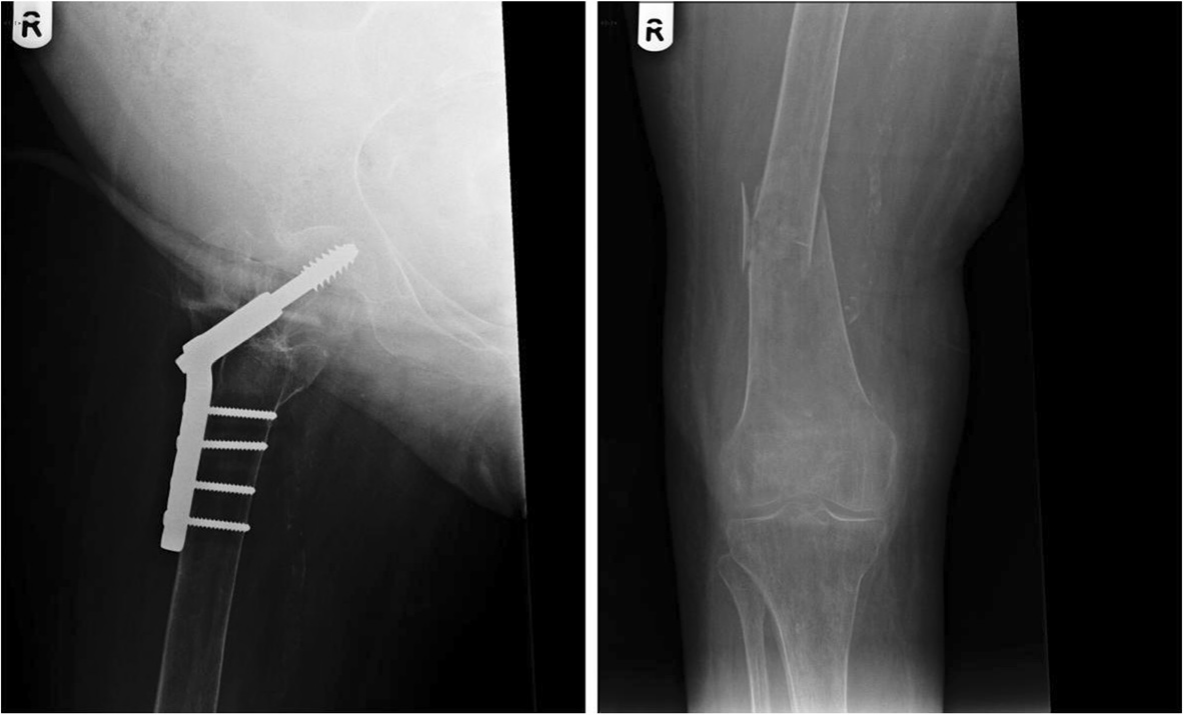
**Left panel: X-ray of right thigh of patient #3 revealed previous dynamic hip screw internal fixation right hip.** Right panel: X-ray of right thigh and knee of patient #3 revealed fracture distal third shaft of femur with mild varus angulation.

A 91-years-old lady had paraplegia, sustained as a result of vascular accident which occurred 24 years previously. She had been managing her bladder by indwelling urethral catheter. Suprapubic cystostomy was performed when she was eighty years old. This patient did not develop stones in urinary bladder; there was no history of passing blood in urine. Ultrasound examination of urinary tract, performed in 2011, revealed no hydronephrosis. In 2013, at the age of 91 years, this patient underwent routine ultrasound examination of urinary tract. This patient did not pass blood in urine; she did not get urine infections. There was no problem with suprapubic catheter. Ultrasound scan was performed by a Consultant Radiologist, who reported a large 4 cm × 3 cm soft tissue mass in the urinary bladder. (Figure 3) Appearances were highly suggestive of urinary bladder transition cell carcinoma. Urine cytology showed benign epithelial cells, scattered columnar cells, mixed inflammatory cells and background bacteria. No malignant cells were seen. Cystoscopy was performed without anaesthesia in lithotomy position. Positioning was challenging, but no untoward incident was noticed in the operation theatre. Cystoscopy revealed normal bladder mucosa; no stones; no tumour. This patient was discharged home three days after she underwent cystoscopy. When she returned to her house, her daughter noticed that right knee was swollen and there was deformity of lower third of right thigh. This patient was admitted to a hospital for assessment of mild swelling of whole of right leg, particularly, knee. Diagnosis was “right leg swelling - osteo-arthritis of knee”. The swelling was non-tender, tense; no erythema was present. Doppler scan of right leg revealed no evidence of deep vein thrombosis. X-ray of right thigh or knee was not taken. This patient was advised that Computed Tomography of abdomen and pelvis would be considered as outpatient if the swelling did not settle or became worse. This patient stayed in bed at home. But the swelling did not subside and deformity of lower third of thigh became more obvious. Therefore, this patient was brought to spinal unit by her daughter. X-ray of right thigh and knee revealed previous dynamic hip screw internal fixation right hip (Figure 4); comminuted fracture distal third of femur with mild lateral displacement and moderate anterior angulation. (Figure 4) Orthopaedic surgeon advised immobilisation of right lower extremity in a plaster cast and decided against surgical fixation of fractured femur. This patient was prescribed Enoxaparin 40 mg subcutaneously as prophylaxis for deep vein thrombosis. Blood test revealed vitamin D deficiency (total serum 25 hydroxy vitamin D level: 26 nmol/L; reference range: 50–150). Calcium: 2.41 mmol/L (reference range: 2.20 - 2.60); Alkaline phosphatase: 175 U/L (reference range: 27–107). This elderly patient was prescribed Colecalciferol 20,000 units daily for 15 days to be taken with the main meal of the day, followed by Colecalciferol 800 units daily.

Follow-up ultrasound examination of urinary bladder and kidneys was performed by a senior Consultant Radiologist four weeks later. Ultrasound scan revealed normal ultrasound appearance of the kidneys with no hydronephrosis and no obvious renal calculi. Suprapubic catheter was in right position. Outline of urinary bladder was normal; there was no neoplastic filling defect or calculus. (Figure 3) Previous ultrasound examination was performed without filling the bladder with sterile 0.9% sodium chloride solution. Most probably, the appearance of a tumour during previous ultrasound examination was caused by a collapsed bladder folding around the balloon of Foley catheter.

Patient’s daughter had to rush home from her holidays abroad when the initial ultrasound examination reported presence of bladder tumour. Luckily cystoscopy revealed no tumour, but this elderly patient sustained fracture of femur when her lower extremities were placed in lithotomy position for cystoscopy. She required plaster cast, and it was difficult for her to manage at home with the knee in plaster cast in extended position. Therefore she stayed in spinal unit until the fracture healed.

## Discussion

### Substandard care of elderly patients: unrecognised epidemic?

We found that some elderly patients receive substandard care in the hospital as well as in the community. Although a new study shows that optimal emergency care for acute coronary syndrome in the very elderly significantly increases their chances of survival—just as it does in younger patients—it also reveals that optimal care for acute coronary syndrome is often withheld from nonagenarians and centenarians [6,7]. A review of Veterans Administration (VA) pharmacy and patient care records to identify instances of inappropriate prescribing among 850,154 patients, who received care at 124 VA facilities during 1999 and 2000, revealed that, overall, 26.2 percent of elderly patients were given drugs identified as inappropriate or suboptimal for older patients [8].

In United Kingdom, the Care Quality Commission of England has been reported to say that too often, staff appears to pay more attention to paperwork than to those they are looking after. Unacceptable care has become standard in some trusts, with doctors and nurses talking down to patients, ignoring their calls for assistance and failing to help them eat, drink or wash. After carrying out spot checks at geriatric wards in 100 hospitals, the commission found that 35 needed to make improvements, 18 were failing to meet legal standards and there were “major concerns” at two trusts. Its report is the latest to conclude that pensioners, who account for almost half of in-patients, are routinely denied the most basic care because of a culture of neglect among staff. In some places, elderly patients were left rattling their bed rails or hitting water jugs on tables to attract nurses’ attention [9,10]. *In the three cases reported here, a common link seems to be failure to recognise the complications promptly by the health professionals, who carried out the procedures. Similarly, failure to seek advice and expert opinion from senior colleagues appears to be another problem*.

### How to prevent substandard care of elderly patients?

In patient #3, a collapsed bladder was misdiagnosed as bladder tumour by a Consultant Radiologist. Misdiagnosis of bladder tumour during ultrasound examination is very rare. DeCaro and associates reported the case of a woman treated with dextranomer-hyaluronic acid for childhood vesicoureteral reflux misdiagnosed with a bladder tumor on transvaginal ultrasonography [11]. In the past, we have misdiagnosed bladder tumour as vesical debris in a paraplegic patient [12]. *We learn that frequent, informal and honest discussions of a patient’s clinical condition, is likely to reduce urological errors in spinal cord injury patients*[13]*. In this elderly patient, such a honest discussion with a senior colleague would have prevented the misdiagnosis of bladder tumour and stopped this elderly patient from undergoing cystoscopy unnecessarily.*

### Most importantly, elderly patients should be treated by senior, experienced doctors and nurses

Quite often, elderly patients may be used as “guinea pigs” for learning by trainee doctors and student nurses because many elderly patients are very polite and are not assertive. Whereas a trainee doctor or a student nurse is unlikely to practise their clinical skills on a rich banker, who is very assertive, an elderly patient who is docile and hard of hearing, often “agrees” to be seen by a junior doctor or a student nurse. It is interesting to note that none of the three patients complained about the substandard medical care, which they received. These elderly patients accepted graciously the consequences of substandard care; such behaviour probably reflects upon their upbringing and the moral code, which many elderly people practise.

### Professional requirements of a doctor when elderly patients receive substandard care

When elderly patients receive substandard care, patients should be told the truth; doctors should express regret, and give information on how similar outcomes will be prevented in the future [14]. The Medical Protection Society also supports the principles of full and open communication with a patient after a serious adverse outcome including an apology [15]. A wall of silence after an adverse incident can compound mistrust, and provoke formal complaints and legal action. If it is clear that something has gone wrong, an apology is called for, and it should be forthcoming. Contrary to popular belief, apologies tend to prevent formal complaints rather than the reverse. An open and honest explanation and an apology where appropriate, may be what are needed to reassure a patient and avoid any escalation. *Doctors should resist the desire to prove they are “right”, which can lead to an escalation of emotion and ultimately an argument.* Doctors should express empathy and avoid an argument. “Winning an argument” is not the same as achieving a good outcome.To epitomise *“Patients don’t care how much you (doctors) know until they know how much you (doctors) care”*[16]*.*

### Take home message

•Elderly spinal cord injury patients should be treated by senior, experienced doctors, who are familiar with changes in body systems due to old age, compounded further by spinal cord injury. In all the cases reported here, procedures were performed by junior, inexperienced nurses or doctors and mistakes occurred to the detriment of patients.

•When an adverse outcome occurs, the General Medical Council in United Kingdom state that it is a professional requirement of a doctor to put matters right (if that is possible); offer an apology; explain fully and promptly what has happened. Patients and especially, their relatives want information on how similar outcomes will be prevented in the future. The National Health Service of United Kingdom commits to ensure that when mistakes happen, patients receive an appropriate explanation and apology.

## Competing interests

The authors declare that they have no competing interests.

## Authors’ contributions

SV performed flexible cystoscopy in patient #1, managed all patients, conceived the idea for this manuscript, collected the data, wrote the draft and revised the manuscript as per editorial comments. GS performed cystoscopy in patient #3; BS was Consultant in charge of three patients; PH carried out follow-up ultrasound examination of patient # 3; KP assessed cardiac status of patient #3. All authors have been involved in drafting the manuscript, revising it critically for important intellectual content, and have given final approval of the version to be published.
